# Immobilization of *Bacillus amyloliquefaciens* SP1 and its alkaline protease in various matrices for effective hydrolysis of casein

**DOI:** 10.1007/s13205-016-0519-2

**Published:** 2016-09-27

**Authors:** Shiwani Guleria, Abhishek Walia, Anjali Chauhan, C. K. Shirkot

**Affiliations:** 1 Department of Microbiology, Lovely Professional University, Phagwara, Punjab India; 2 Department of Mirobiology, DAV Uiversity, Jalandhar, Punjab India; 3 Department of Basic Sciences, Dr. Y. S. Parmar University of Horticulture and Forestry, Nauni, Solan, HP India

**Keywords:** Immobilization, Polyacrylamide, Calcium alginate, Agar, *Bacillus amyloliquefaciens*

## Abstract

An extracellular alkaline protease producing *B. amyloliquefaciens* SP1 was isolated from apple rhizosphere having multifarious plant growth-promoting activities. *B. amyloliquefaciens* SP1 protease was immobilized using various concentrations of calcium alginate, agar and polyacrylamide to determine the optimum concentration for formation of the beads. Enzyme activity before immobilization (at 60 °C, pH 8.0 for 5 min) was 3580 µg/ml/min. The results of immobilization with various matrices revealed that 3 % calcium alginate (2829.92 µg/ml/min), 2 % agar (2600 µg/ml/min) and 10 % polyacrylamide (5698.99 µg/ml/min) were optimum concentrations for stable bead formation. Immobilized enzyme reusability results indicated that calcium alginate, agar and polyacrylamide beads retained 25.63, 22.05 and 34.04 % activity in their fifth repeated cycle, respectively. In cell immobilization technique, the free movement of microorganisms is restricted in the process, and a semi-continuous system of fermentation can be used. In the present work, this technique has been used for alkaline protease production using different matrices. Polyacrylamide (10 %) was found with the highest total alkaline protease titer, i.e., 24,847 µg/ml/min semi-continuously for 18 days as compared to agar (total enzyme titer: 5800 in 10 days) and calcium alginate (total enzyme titer: 13,010 in 15 days). This present study reported that polyacrylamide (10 %) among different matrices has maximum potential of immobilization of *B. amyloliquefaciens* SP1 and its detergent stable alkaline protease with effective application in bloodstain removal.

## Introduction

Proteases (EC 3.4) are one of the largest and most diverse families of enzymes known till date. These hydrolyze proteins into short peptides or free amino acids. Proteases do not refer to a single enzyme, but a mixture of enzymes including proteinases and amidases (Qureshi et al. 2011). These enzymes have versatile applications in various industries, such as detergents, food, pharmaceutical, leather, silk, silver recovery and production of protein hydrolysates (Annamalai et al. 2013). Proteases are also envisaged as having extensive applications in the development of eco-friendly technologies as well as in several bioremediation processes (Jisha et al. 2013; Wang et al. 2009). Microbial proteases constitute one of the most important groups of industrial enzymes and their dominance in the industrial market is expected to increase further in coming years. Among different types (acidic, neutral and alkaline) of proteases, alkaline proteases are the most commonly used industrial enzyme in view of their activity and stability at high pH (Denizci et al. 2004; Devi et al. 2008).

Microbial proteases are usually produced by either free or immobilized cells. Immobilized whole cell/enzyme production offers several advantages such as better operational stability due to biocatalyst and high efficiency of catalysis. Immobilization of an enzyme allows for easy separation of the enzyme from the products and for recycling of the enzyme (Demirkan et al. 2011). Immobilized cells are used in food technology, biotechnology, biomedicine and analytical chemistry (Kumar and Vats 2010). It offers various advantages, such as increase of productivity due to the high cell concentration within the reactor, prevention of washout in continuous operation among others (Sankaralingam et al. 2012) and the ability to separate cell mass from the bulk liquid for possible reuse (Kumar and Vats 2010). However, proper selection of immobilization techniques and supporting materials is needed to minimize the disadvantages of immobilization. Generally, enzymes or cells are immobilized by physical adsorption, ionic binding, covalent binding, cross-linking and entrapment methods. Entrapment method has been considered most suitable among the various reported methods. It consists of enclosing the enzyme or cell in an aqueous solution inside a semi-permeable membrane capsule. It is possible to immobilize several enzymes or cells at the same time (Demirkan et al. 2011). Thus in the present study, the potential of various matrices for immobilization of alkaline protease and *B. amyloliquefaciens* SP1 was investigated.

## Materials and methods

### Bacterial strain and growth conditions

Previously isolated *B. amyloliquifaciens* SP1 (Accession Number KF923792) from apple rhizosphere of Chamba District, Himachal Pradesh, India, was used. It has the potential to produce detergent-stable alkaline protease along with other plant growth-promoting activities. Strain SP1 was grown using a production medium (pH 8.0) having the composition g/100 ml: 2 g casein, 0.4 g yeast extract, 2 g maltose, 0.1 g KH_2_PO_4_ and 2 g gelatin at 37 °C for 48 h (Guleria et al. 2014, 2015, 2016). The culture filtrate was used for further studies as enzyme source.

### Analytical methods

#### Determination of proteolytic activity

The cell-free supernatant was used for protease assay, and the enzyme activity was measured according to the method described by Sigma Aldrich with slight modifications (Enyard 2008). The reaction mixture containing 1 ml of enzyme was added to 5 ml of 0.5 % casein solution in 0.2 M tris–HCl buffer of pH 8.0 and the mixture was incubated for 5 min at 60 °C. The reaction was terminated by adding 5 ml of trichloroacetic acid (110 mM) and kept for 30 min at room temperature and then centrifuged for 5 min at 10,000 rpm. Then 2 ml of filtrate was mixed with 5 ml of 500 mM Na_2_CO_3_ solution followed by 1 ml Folin–Ciocalteu reagent. The amount of tyrosine released was determined spectrophotometrically at 660 nm against the enzyme blank. The control was treated in the same way, except that TCA was added before the addition of enzyme solution.

One unit of protease activity was equivalent to the amount of enzyme that required releasing 1 µg of tyrosine/ml/min under standard assay conditions.

#### Protein determination

The concentration of protein in the culture supernatant was estimated using the Lowry’s method (Lowry et al. 1951) of protein estimation with bovine serum albumin as a standard.

### Entrapment methods for immobilization

#### Calcium alginate immobilization

The sodium alginate suspension was prepared by gradually adding a small amount of 0.9 g sodium alginate into 30 ml of boiling water under constant stirring, autoclaved at 121 °C for 15 min and allowed to cool to room temperature (Adinarayana et al. 2005). For whole cell immobilization, 250 µl of 24-h-old shake culture was added and mixed for 10 min by stirring. For enzyme immobilization, 1 ml crude enzyme was added to 9 ml of sodium alginate suspension (1, 2 and 3 %) and thoroughly mixed. The resulting preparation was taken in a sterile syringe and added dropwise into chilled 0.2 M CaCl_2_ solution from 5 cm height with constant stirring. The beads obtained were kept for curing at 4 °C for 1 h in a refrigerator. The cured beads so formed were washed two to three times with sterile distilled water and stored at 4 °C until use. All the experimentations were carried out aseptically in a laminar flow hood.

#### Agar immobilization

For enzyme immobilization, 1 ml of crude enzyme was mixed with 1, 2 and 3 % of autoclaved (121 °C for 15 min) and cooled 9 ml agar solution (Adinarayana et al. 2005). For whole cell immobilization, 250 µl of 24-h-old shake culture was added to 30 ml molten agar maintained at room temperature and poured into sterile flat bottom Petri plates. The solidified agar blocks were cut into equal size cubes and added to sterile 20 mM Tris–HCl buffer (pH 8.0) and kept at 4 °C for 1 h. After curing, Tris–HCl buffer was decanted and cubes were washed with sterile distilled water three to four times and stored at 4 °C until use.

#### Polyacrylamide immobilization

Polyacrylamide solution was prepared by mixing acrylamide (30 %) and bisacrylamide (0.8 %), and to this ammonium persulfate (10 %) was added. For enzyme immobilization, 1 ml of crude enzyme was mixed with 8, 10 and 12 % sterile 9 ml polyacrylamide solution, and for whole cell immobilization, 250 µl of 24-h-old culture was added to the polyacrylamide solution (Adinarayana et al. 2005). Further, 5 µl TEMED was mixed to the resulting preparations for polymerization and poured into flat-bottom Petri plates for solidification. After polymerization, polyacrylamide gel was cut into equal size cubes. The cubes were transferred to 20 mM Tris–HCl buffer (pH 8.0) and kept at 4 °C for curing for 1 h. The cubes were washed with sterile distilled water and stored at 4 °C until use. All experimentations were carried under aseptic conditions.

#### Reusability of immobilized enzyme

Enzyme immobilization was done with different concentrations of different matrix types, viz., agar (1, 2 and 3 %), calcium alginate (1, 2 and 3 %) and polyacrylamide gel (8, 10 and 12 %). To determine the reusability of the immobilized enzyme of different matrices, beads were removed and washed after every use with distilled water and added into fresh reaction mixture for 5 min at 60 °C. Alkaline protease activity was measured by standard protease assay method.

### Whole cell immobilization

#### Optimization of incubation time for alkaline protease production in liquid medium by immobilized cells

Whole cell immobilization was done with different matrices, viz., agar (2 %), calcium alginate (3 %) and polyacrylamide (10 %) gel. The batch experiment was performed in 250 ml capacity Erlenmeyer flask containing 25 ml production medium of pH 8.0. The immobilized beads of different matrices were transferred to the production medium. The flasks were incubated at 37 °C up to 96 h on a shaker incubator at 100 rpm. Samples were withdrawn at regular intervals of 12 h from each flask. Enzyme activity and cell leakage population of each sample were determined.

#### Reusability of immobilized cells

To determine the reusability of the immobilized cells of different matrices, repeated batch fermentations were conducted with optimized culture conditions. In the repeated batch process, beads were removed from the production medium after optimum incubation time, washed with 0.9 % NaCl and added into fresh sterile production medium for the next production cycle (Adinarayana et al. 2005). The reusability of immobilized cells was continued until no alkaline protease activity was detected in the cell-free supernatant.

#### Application of immobilized protease on bloodstain removal

Immobilized protease was investigated as a detergent additive. For this, white cotton cloth pieces (10 cm × 10 cm) were stained with blood and oven dried at 95–100 °C for 5 min. The stained cloth pieces were taken in separate trays. The following sets were prepared and studied:

1. Tray with distilled water (100 ml) + bloodstained cloth (control).
2. Tray with distilled water (100 ml) + bloodstained cloth + 1 ml of commercial detergent (1 % Ariel).
3. Tray with distilled water (100 ml) + bloodstained cloth + Polyacrylamide immobilized enzyme (2 ml crude enzyme).
4. Tray with distilled water (100 ml) + bloodstained cloth + 1 ml of commercial detergent (1 % Ariel) + Polyacrylamide-immobilized enzyme (2 ml crude enzyme).

The trays were incubated at 60 °C for 30 min. The cloth pieces were taken out from each set at regular intervals of 5 min, rinsed with water, dried and visually examined. Untreated cloth pieces stained with blood was considered as control.

## Results

### Enzyme immobilization

#### Effect of sodium alginate concentration on alkaline protease activity and reusability of immobilized enzyme

The effect of sodium alginate concentration ranging from 1 to 3 % (w/v) on alkaline protease activity and reusability of entrapped enzyme is shown in Table 1. The data confirmed that significantly higher protease activity (2829.92 µg/ml/min) of entrapped enzyme was obtained with beads prepared with 3 % (w/v) sodium alginate. Thereafter, a gradual decrease in alkaline protease activity with decrease in concentration of sodium alginate was observed. The lowest alkaline protease activity (2525.22 µg/ml/min) was observed with 1 % sodium alginate.

**Table 1:** Effect of calcium alginate concentration (1–3 %) on immobilization of *B. amyloliquefaciens* SP1 alkaline protease and reusability of immobilized enzyme

Cycle (5 min each)	Protease activity (µg/ml/min)		
	Sodium alginate concentration (%)		
	1	2	3
1	2525.22	2741.00	2829.92
2	2507.00	2394.58	2757.42
3	1321.12	1636.50	1730.70
4	930.30	984.40	1212.56
5	631.86	702.51	982.93
lsd	11.53	8.44	8.44

The reusability of entrapped enzyme in different concentrations of sodium alginate (1–3 %) was evaluated by transferring the entrapped enzyme to the fresh reaction mixture at pH 8.0 and temperature 60 °C for 5 min. The results showed that the entrapped enzyme prepared with 1–3 % (w/v) sodium alginate could be reused effectively for five cycles. It was observed that the amount of protease activity with 3 % calcium alginate-entrapped enzyme was decreased significantly from 2829.92 µg/ml/min in the first cycle to 982.93 µg/ml/min in the fifth reuse cycle. Similarly, a gradual decrease in alkaline protease activity from 2525.22 to 631.86 µg/ml/min and 2741 to 702.51 µg/ml/min was observed in 2 and 1 % alginate-entrapped enzyme. The differences in alkaline protease activity at various repeated cycles of 1, 2 and 3 % alginate entrapped enzyme were statistically significant except for the first and second cycle of 1 % alginate-entrapped enzyme.

#### Effect of agar concentration on alkaline protease activity and reusability of immobilized enzyme

Perusal of data appended in Table 2 revealed the effect of agar concentration ranging from 1 to 3 % on alkaline protease activity and reusability of entrapped enzyme. It was observed that beads prepared with 2 % (w/v) agar produced significantly higher (2600.00 µg/ml/min) protease activity. There was significant decrease in the entrapped enzyme activity with increase or decrease in the concentration of agar. The lowest alkaline protease activity (2431.74 µg/ml/min) was observed with 1 % agar beads.

**Table 2:** Effect of agar concentration (1–3 %) on immobilization of *B. amyloliquefaciens* SP1 alkaline protease and reusability of immobilized enzyme

Cycles (5 min each)	Protease activity (µg/ml/min)		
	Agar concentration (%)		
	1	2	3
1	2431.74	2600.00	2542.88
2	2126.67	2420.00	2431.74
3	906.01	1324.00	1118.72
4	388.24	610.00	526.98
5	295.87	573.27	490.18
lsd	6.90	10.14	6.88

The reusability of entrapped enzyme in different concentrations of agar was determined by transferring the entrapped enzyme into a fresh reaction mixture at 60 °C, pH 8.0 for 5 min. The results showed that entrapped enzyme prepared with 1–3 % (w/v) agar could be reused effectively for five cycles. The minimum loss of alkaline protease activity with 2 % agar-entrapped enzyme was observed that ranged from 2600.00 µg/ml/min in the first cycle to 573.27 µg/ml/min in the fifth reuse cycle. Similarly, a gradual decrease in alkaline protease activity in agar-entrapped enzyme from 2431.74 to 295.87 µg/ml/min and 2542.88–490.18 µg/ml/min was observed in 1 and 3 %, respectively. The differences in alkaline protease activity at various repeated cycles of 1, 2 and 3 % agar-entrapped enzyme were statistically significant.

#### Effect of polyacrylamide concentration on alkaline protease activity and reusability of immobilized enzyme

The effect of polyacrylamide concentration ranging from 8 to 12 % on alkaline protease activity and reusability of entrapped enzyme is shown in Table 3. The results indicated that the maximum entrapped protease activity (5698.99 µg/ml/min) was obtained with beads prepared with 10 % (w/v) polyacrylamide. A further increase or decrease in polyacrylamide concentration significantly decreased the entrapped protease activity. However, the minimum entrapped alkaline protease activity (3647 µg/ml/min) was observed with 12 % polyacrylamide.

**Table 3:** Effect of polyacrylamide concentration (8–12 %) on immobilization of *B. amyloliquefaciens* SP1 protease and reusability of immobilized enzyme

Cycles (5 min each)	Protease activity (µg/ml/min)		
	Polyacrylamide concentration (%)		
	8	10	12
1	4552.00	5698.99	3647.00
2	3047.00	4047.00	2940.00
3	1964.00	2988.00	1045.00
4	1440.00	2447.00	957.00
5	964.00	1939.90	758.00
lsd	6.90	4.96	6.91

The ability of entrapped enzyme reused at different concentrations of polyacrylamide (8–12 %) was determined by transferring the entrapped enzyme to a fresh reaction mixture at 60 °C, pH 8.0 for 5 min. It was revealed that the entrapped enzyme prepared with 8–12 % (w/v) polyacrylamide could be reused effectively for five cycles. The result revealed that the maximum loss of alkaline protease activity was observed in 10 % polyacrylamide-entrapped enzyme that ranged from 5698.99 to 1939.9 µg/ml/min, whereas the minimum loss of alkaline protease activity was observed with 12 % polyacrylamide-entrapped enzyme that ranged from 3647 µg/ml/min in the first cycle to 758 µg/ml/min in the fifth reuse cycle. However, at various repeated cycles of polyacrylamide-entrapped enzyme, a statistically significant difference in the alkaline protease activity was observed (8, 10 and 12 %).

#### Comparison of the free enzyme system with immobilized enzyme in various matrices for total protease activity

A comparison of the total alkaline protease activity of entrapped enzyme in various matrices (Table 4) revealed that the maximum total alkaline protease activity was 17,120.89 µg/ml/min for polyacrylamide (10 %)-entrapped enzyme, which was found to be significantly higher than that of other immobilized systems and free enzymes. However, among different immobilized matrices, the minimum total alkaline protease activity, i.e., 7527.27 µg/ml/min, was noticed with agar (2 %)-entrapped enzyme. On the basis of total alkaline protease activity, polyacrylamide matrix was found to be superior as compared to the free enzyme system and other matrices types. However, relative to the free enzyme system, 478.24, 265.74 and 210.26 % activity was observed in polyacrylamide- (10 %), calcium alginate- (3 %) and agar (2 %)-immobilized enzyme, respectively. Results revealed that the maximum entrapped enzyme activity per batch was also observed for polyacrylamide-entrapped enzyme (3424.18 µg/ml/min), which was significantly higher than that of agar-entrapped enzyme (1505.45 µg/ml/min) and calcium alginate-entrapped enzyme (1902.71 µg/ml/min). In contrast, free enzyme activity was 3580 µg/ml/min.

**Table 4:** Comparison of alkaline protease activity of enzyme immobilized in various matrices

Matrix	Total alkaline protease activity (µg/ml/min)	Activity relative to free enzyme system (%)	Average protease activity per batch (µg/ml/min)
Polyacrylamide (10 %)	17,120.89	478.24	3424.18
Calcium alginate (3 %)	9513.53	265.74	1902.71
Agar (2 %)	7527.27	210.26	1505.45
Free enzyme (conventional)	3580.00	100.00	3580.00
lsd	6.89	0.69	4.91

### Whole cell immobilization

#### Alkaline protease production by calcium alginate (3 %)-immobilized cells

**Fig. 1:**
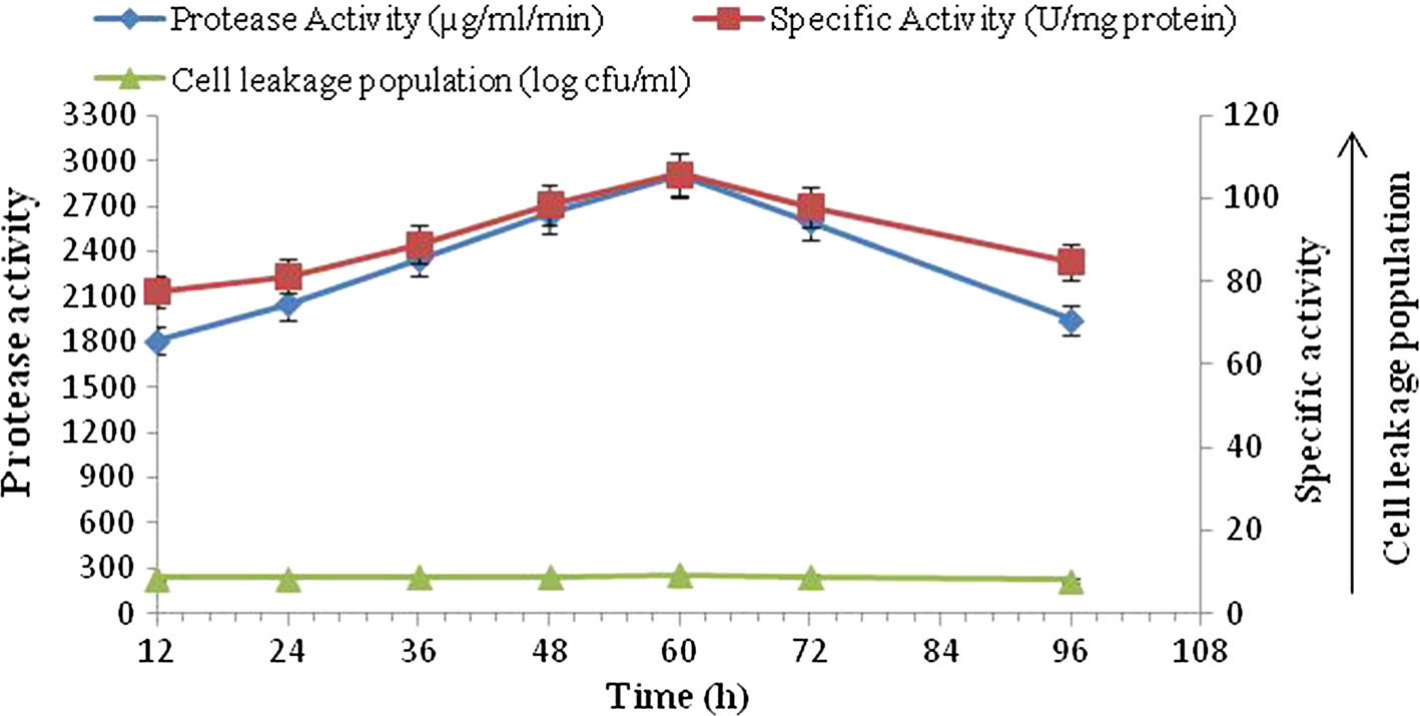
Time course profile of alkaline protease production, specific activity and cell leakage population by immobilized culture of *B. amyloliquefaciens* SP1 in calcium alginate (3 %)

The production of protease by calcium alginate-immobilized cells of *B. amyloliquefaciens* SP1 is shown in Fig. 1. The enzyme production started at 12 h of incubation and gradually increased to reach a maximum level of 2900 µg/ml/min at 60 h of incubation. After this, the enzyme production decreased to 1950 µg/ml/min at 96 h of the incubation period. The production of protease was found to be associated with the population of cells in the medium, resulting from the multiplication of the number of immobilized cells leaked from the beads, i.e., cell leakage population which increased up to 60 h (9.33 log cfu/ml), further increase in incubation period resulting in decrease in cell leakage population (8.08 log cfu/ml) and, therefore, decrease in alkaline protease activity. The correlation coefficient (*r* = 0.76) revealed that there was strong positive correlation between protease production with cell leakage population. However, at various incubation periods, a statistically significant difference in the alkaline protease activity and cell leakage population was observed. The results revealed that the total protein (27.4 mg/ml) and enzyme activity concomitantly increased up to 60 h along with specific activity (105.84 U/mg protein).

#### Alkaline protease production by polyacrylamide (10 %)-immobilized cells

**Fig. 2:**
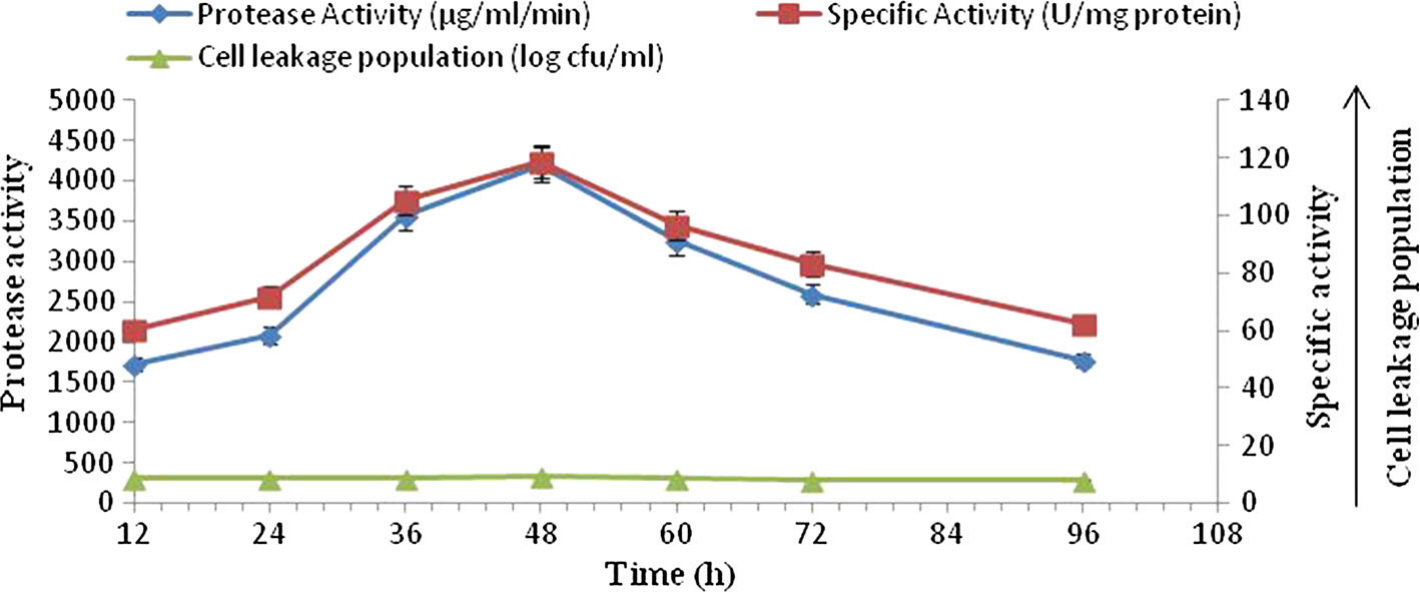
Time course profile of alkaline protease production, specific activity and cell leakage population by immobilized culture of *B. amyloliquefaciens* SP1 in polyacrylamide (10 %)

The production of proteolytic activity was monitored for 96 h in polyacrylamide-entrapped cells at pH 8.0 and 37 °C. The results indicated that the cell leakage population and alkaline protease production increased with a prolonged incubation period up to 48 h. A statistically significant gradual increase in alkaline protease production was noticed from 12 h onward to 48 h; on further incubation, a decline in alkaline protease activity was observed (Fig. 2). The difference in alkaline protease production and cell leakage population obtained at various incubation periods was found to be statistically significant. A positive correlation coefficient (*r* = 0.63) was found between protease production and the cell leakage population.

**Fig. 3:**
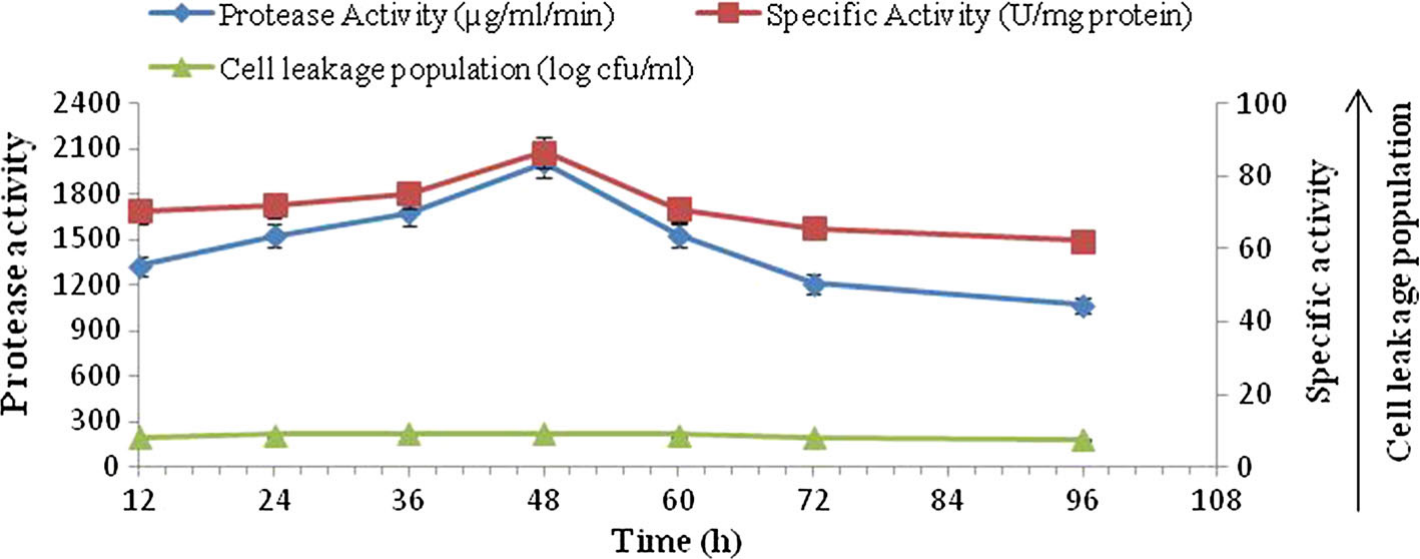
Time course profile of alkaline protease production, specific activity and cell leakage population by immobilized culture of *B. amyloliquefaciens* SP1 in agar (2 %)

The maximum alkaline protease activity of 4200 µg/ml/min was observed at 48 h with maximum specific activity (118.64 U/mg protein) and cell leakage population (9.25 log cfu/ml). Maximum protease production was observed with polyacrylamide (10 %)-entrapped cells as compared to free cell system (3450 µg/ml/min), alginate (2900 µg/ml/min) and agar-entrapped cells (2010 µg/ml/min) (Figs. 1, 3).

#### Alkaline protease production by agar (2 %)-immobilized cells

A study on alkaline protease production by agar-immobilized cells of *B. amyloliquefaciens* SP1 was conducted by incubating the flasks for 96 h at 37 °C and pH 8.0 (Fig. 3). The data indicated that alkaline protease production started after 12 h (1324.88 µg/ml/min), reached a maximum level by 48 h (2010 µg/ml/min) and, thereafter, gradually decreased to 1070 µg/ml/min at the end of 96 h incubation period. The production of alkaline protease was found to be associated with cell leakage population which increased up to 48 h (9.25 log cfu/ml), a further increase in incubation period resulting in decrease in cell leakage population and, therefore, decrease in alkaline protease activity. The statistical analysis revealed that the correlation coefficient between alkaline protease production and cell leakage population was positive (*r* = 0.94). The difference in alkaline protease production and cell leakage population obtained at various incubation periods was found to be statistically significant. It was also found that the alkaline protease production with agar-immobilized cells was comparatively lesser than the free cell system (3450 µg/ml/min) and immobilized cells of other matrices (Figs. 1, 2).

There was significant increase in the specific activity (U/mg protein) with increase in the incubation period up to 48 h and thereafter decreased.

#### Repeat batch fermentation of immobilized cells

The semi-continuous fermentation was terminated to investigate the stability of immobilized cells and their ability to produce alkaline protease under repeated batch cultivation conditions. The optimum incubation period standardized earlier for maximum enzyme production was selected for repeated reuse of inoculated beads for all the experiments.

#### Reusability of calcium alginate (3 %)-immobilized cells

**Fig. 4:**
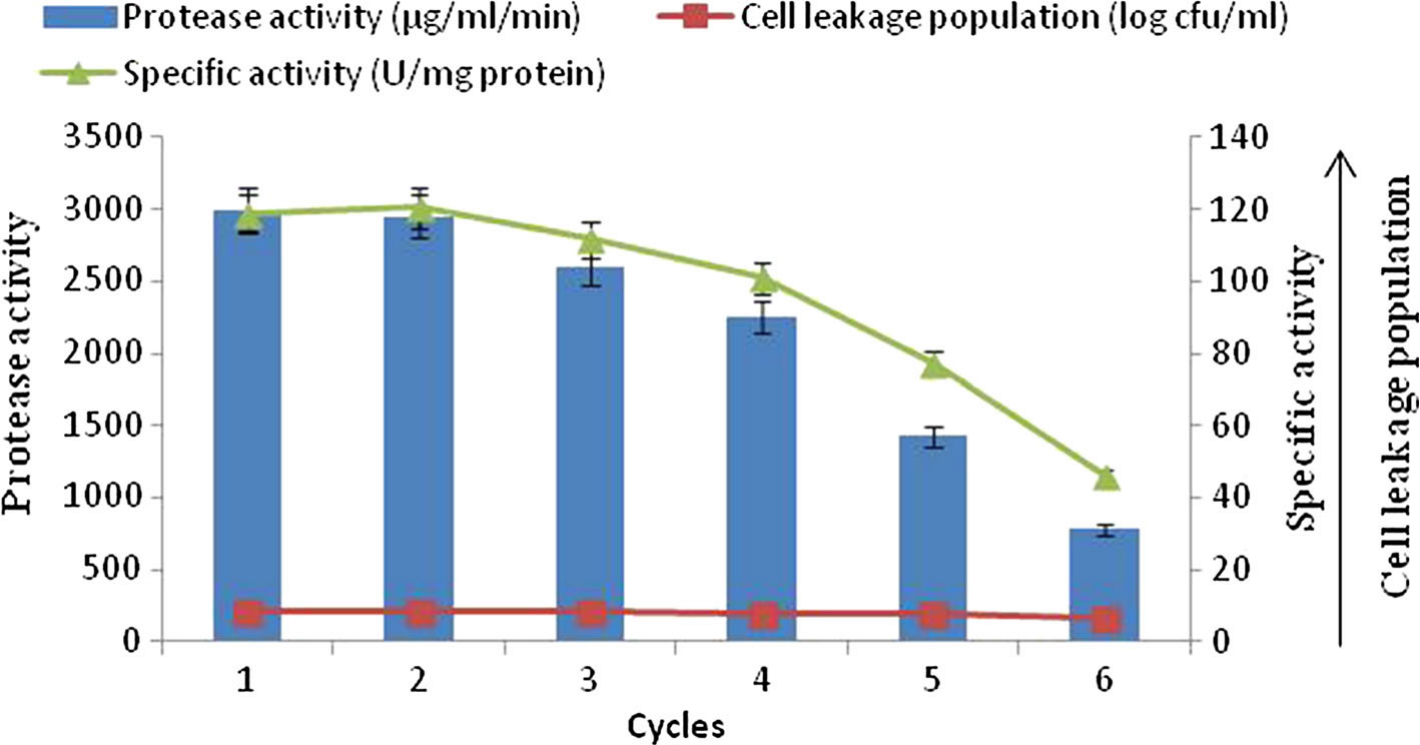
Alkaline protease production by repeated batch culture using immobilized cells of *B. amyloliquefaciens* SP1 in calcium alginate

The reusability of *B. amyloliquefaciens* SP1 was evaluated by transferring the calcium alginate-immobilized cells to fresh medium at every 60 h of incubation. The results (Fig. 4) showed that the alginate-entrapped cells can be reused for a maximum of 15 days (6 cycles), after which the beads were completely disintegrated. The results revealed that the amount of enzyme production with immobilized cells was decreased significantly from 3000 µg/ml/min in the first batch to 780 µg/ml/min in the sixth batch. At the same time, a gradual decrease in the cell leakage population (8.70–6.75 log cfu/ml) and specific activity (111.74–39.39 U/mg protein) was found up to the sixth batch. The correlation coefficient (*r* = 0.95) revealed there was strong positive correlation between alkaline protease production with cell leakage population.

The difference in alkaline protease production and cell leakage population obtained at various repeated batches was statistically significant. However, the specific activity of the first and second batch of fermentation was found to be statistically at par with each other and after that significant decline was observed.

#### Reusability of polyacrylamide (10 %)-immobilized cells

**Fig. 5:**
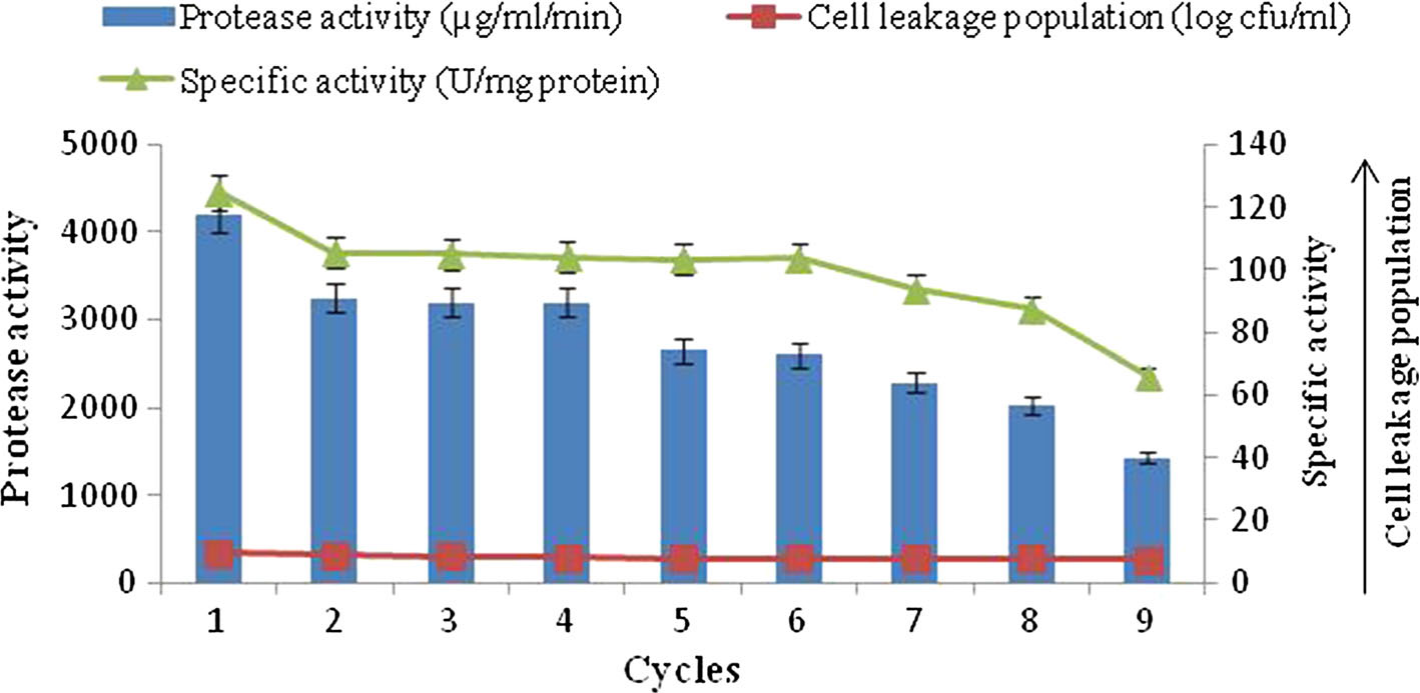
Alkaline protease production by repeated batch culture using immobilized cells of *B. amyloliquefaciens* SP1 in polyacrylamide

The reusability of polyacrylamide-immobilized *B. amyloliquefaciens* SP1 was investigated by transferring the immobilized cells to fresh medium at every 48 h of incubation (Fig. 5). The results revealed that the polyacrylamide-entrapped cells could be reused for a maximum of 18 days (9 cycles) with gradual decrease in the level of alkaline protease production from 4200 µg/ml/min in the first batch to 1430 µg/ml/min in the ninth batch. It was found that immobilized beads were not disintegrated even after the ninth batch, although the production of alkaline protease was decreased significantly.

At the same time, a gradual decrease in the cell leakage population (9.45–7.25 log cfu/ml) was found up to the ninth batch. However, the differences in specific activity of various repeated batches of fermentation were statistically significant among them except from the second to the sixth batch which were found to be statistically at par with each other. A strong positive correlation coefficient was found with alkaline protease production and cell leakage population (*r* = 0.97).

#### Reusability of agar (2 %)-immobilized cells

**Fig. 6:**
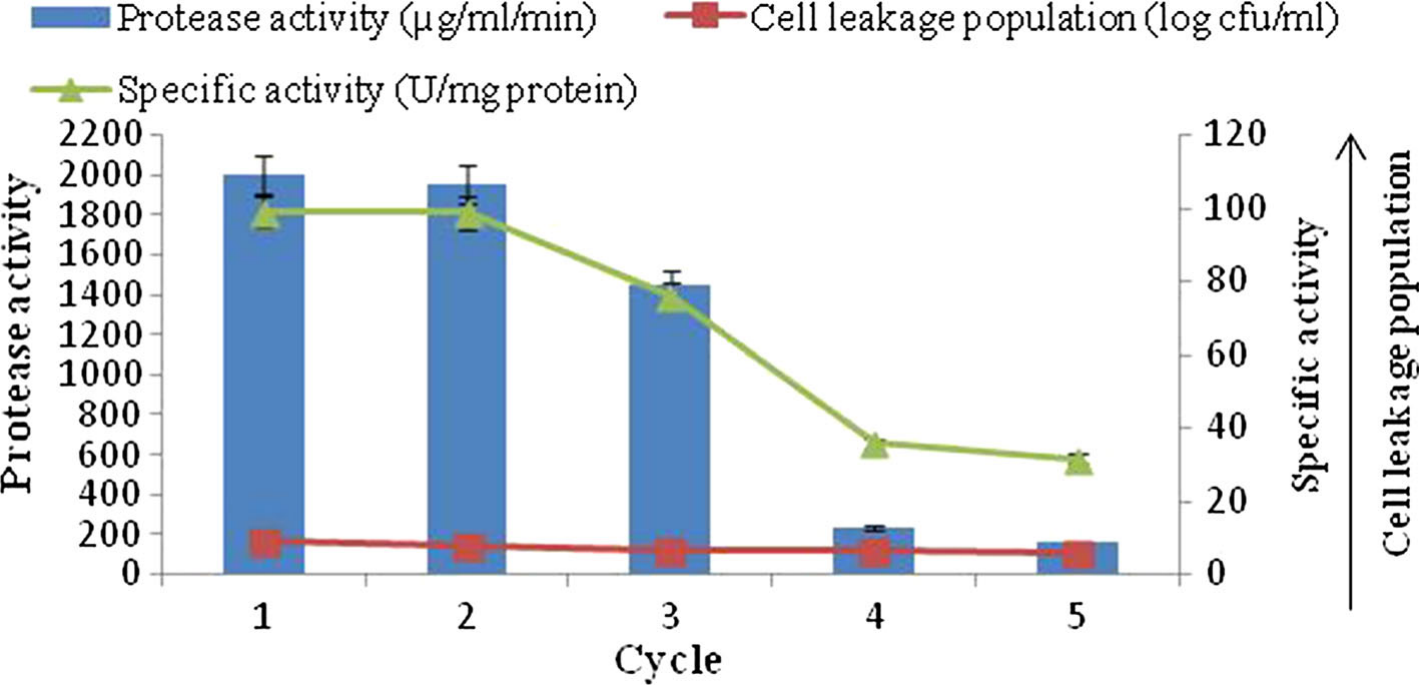
Alkaline protease production by repeated batch culture using immobilized cells of *B. amyloliquefaciens* SP1 in agar

The reusability of agar-entrapped *B. amyloliquefaciens* SP1 was investigated by transferring the immobilized cells to the fresh medium at every 48 h of incubation (Fig. 6). Results revealed that the agar-entrapped cells can be reused for a maximum of 10 days (5 cycles) with gradual decreasing level of alkaline protease production from first batch (2000 µg/ml/min) to fifth batch (160 µg/ml/min). The statistical analysis revealed that the correlation coefficient (*r*) between protease production and cell leakage population was found to be 0.86.

At the same time, significant gradual decrease in cell leakage population (9.25–5.85 log cfu/ml) was found up to the fifth batch. However, specific activity of the first and second batch of fermentation was found to be statistically at par with each other and after that significant decline was observed. Disintegration of beads was not noticed even after the significant loss of activity.

#### Comparison of alkaline protease production by the free cell system with immobilized cells in various matrices for total protease titer

Comparison of total alkaline protease titer of immobilized cells in various matrices (Table 5) revealed that the maximum specific volumetric productivity was 71.88 µg/ml/h for the conventional free cell system that lasted for 48 h and was found to be significantly higher than that of immobilized systems. However, among different immobilized cell matrices, maximum specific volumetric productivity, i.e., 57.52 µg/ml/h was noticed with polyacrylamide-immobilized cells which lasted for 18 days. Similarly, the specific volumetric productivity by repeated batch fermentation was 36.14 µg/ml/h with calcium alginate and 24.17 µg/ml/h with the agar matrix. Results revealed that maximum repeat cycles (9) with the highest total alkaline protease titer (24,847 µg/ml/min) was observed for polyacrylamide-entrapped cells, which was significantly higher than agar-entrapped cells (reused for 5 cycles with total alkaline protease titer of 5800 µg/ml/min) and calcium alginate-entrapped cells (reused for 6 cycles with total alkaline protease titer of 13,010 µg/ml/min). In contrast, free cell fermentation produced 3450 µg/ml/min total alkaline protease titer. The highest average activity per batch among different matrices system was also found in case of polyacrylamide-entrapped cells (2760.78 µg/ml/min) followed by calcium alginate-immobilized cells (2168.33 µg/ml/min).

**Table 5:** Comparison of alkaline protease production with cells immobilized in various matrices

Matrix	Fermentation period for each batch (h)	Number of batches	Total fermentation time (h)	Total alkaline protease titer (µg/ml/min)	Relative activity (%)	Average protease activity per batch (µg/ml/min)	Specific volumetric productivity (µg/ml/h)
Polyacrylamide	48	9	432	24,847.00	100.00	2760.78	57.52
Calcium alginate	60	6	360	13,010.00	52.36	2168.33	36.14
Agar	48	5	240	5800.00	23.34	1160.00	24.17
Free cells (conventional)	48	1	48	3450.00	13.89	3450.00	71.88
lsd				23.06	0.49	14.38	0.23

On the basis of total alkaline protease titer, the polyacrylamide matrix was found to be superior as compared to the free cell system and other matrices type. However, relative to polyacrylamide-entrapped cells, 52.36 and 23.34 % activity was observed in calcium alginate and agar-immobilized cells, respectively, and only 13.89 % relative activity was noticed in conventional free cell system. Hence, polyacrylamide-immobilized cells were able to produce alkaline protease consistently for 18 days.

#### Immobilized protease as detergent additive

The results revealed that bloodstains were completely removed with a combination of polyacrylamide-immobilized enzyme and its most compatible detergent (1 % Ariel) in less than 25 min. However, individual treatment of distilled water, immobilized enzyme and detergent (1 % Ariel) was not able to remove bloodstain even after 30 min.

## Discussion

Use of enzymes as catalysts for large-scale industrial processes is limited by their high cost of production and stabilization on storage. During use, their stability decreases due to changes in pH, temperature, conformational changes as a result of friction, osmotic pressure imposed by the environs on their use, and a cumulative effect of all these factors as a function of duration of their use. Secondly, since they are soluble, their recovery from a mixture of substrate and product for reuse is not economically practical, rendering the costly enzymatic process even more costly (Kotwal and Shankar 2009). Among different techniques, entrapment in agar and calcium alginate gel offers many advantages due to its simplicity and nontoxic character (Gangadharan et al. 2009).

The prerequisites of immobilization of enzyme are proper permeability and rigidity of beads. For this concentration of agar, sodium alginate and polyacrylamide need to be optimized. Data appended in Tables 1, 2 and 3 reveal that the maximum entrapped protease activity was obtained with 2 % agar, 3 % sodium alginate and 10 % polyacrylamide beads. This indicates that alkaline protease activity was minimum when the concentrations of different matrices were low. This may be due to the high permeability of different matrices at low concentration. However, the protease activity decrease at higher concentration of agar (3 %) and polyacrylamide (12 %) was due to decreased gel porosity with increase in gel concentration and, consequently, resulted in diffusion limitation (Ahmed et al. 2010). Similar results were observed previously for other entrapped enzymes (Lamas et al. 2001; Samia and Ahmed 2010).

The reusability of the immobilized enzyme is very important from the point of view of reducing the cost of the enzyme. This is an important factor while considering its suitability for commercial applications (Tanksale et al. 2001). The results of the present work revealed that after five reuses, 25.63, 22.05 and 34.04 % activity was obtained as compared to their first use with calcium alginate-, agar- and polyacrylamide-entrapped enzymes, respectively. This may be due to the leakage of enzyme from the beads during their use and washing at the end of each cycle (Geethanjali and Subash 2013). Anwar et al. (2009) reported entrapment of protease in calcium alginate beads and decrease in protease activity after three reuses. Another study stated that α-amylase entrapped in calcium alginate beads could be reused for six cycles with about 30 % loss in activity (Kumar et al. 2006). Thus, polyacrylamide-entrapped alkaline protease of *B. amyloliquefaciens* SP1 showed very high retaining and reuse ability as compared to that already reported.

Whole cell immobilization is one of the common techniques for increasing the overall cell concentration and productivity. Among the immobilization methods for microbial cells, entrapment is the most suitable and common method. Immobilization by entrapment is known to be a simple and gentle procedure and protects the cells from unfavorable conditions (pH, temperature, etc.) from the surrounding media (Kumar and Vats 2010). Whole cell immobilization technique is generally used for higher productivity by protecting the cells from shear forces. In addition to this, the product and the cell can easily be separated, so that the cells can be reused several times (Adinarayana et al. 2005). Immobilization of cells may allow continuous operation of cultivation at high dilution rates. This strategy protects the cells from shear forces. Immobilization of protease-producing cells has been performed by different authors (Rao et al. 2008; Anwar et al. 2009; Sankaralingam et al. 2012; Geethanjali and Subash 2013).

Different matrices like agar, calcium alginate and polyacrylamide were used for immobilization of a definite amount of logarithm phase, *B. amyloliquefaciens* SP1 cells. Similarly, a free cell system of equal initial cell concentration was also run (Figs. 1, 2, 3). The present investigation revealed the optimum incubation period of different matrices for alkaline protease production. The data indicated that protease production in different matrices started from 24 h and reached maximum at 48 h in case of agar and polyacrylamide matrices, whereas for calcium alginate matrix the maximum alkaline protease production was achieved in 60 h. The results of the present investigation is in disagreement with previous studies (Adinarayana et al. 2005; Kumar and Vats 2010) that showed a higher level of alkaline protease production in 24 h by agar, polyacrylamide and calcium alginate matrices. However, the results are in agreement with those from other investigations that found 48 h as optimum incubation time for these matrices-entrapped cells (Sankaralingam et al. 2012). The production of alkaline protease was found to be associated with the population of the cells in the medium, resulting from the multiplication of the number of immobilized cells leaked from the beads, i.e., cell leakage population which increased up to 48 h in case of agar and polyacrylamide immobilization and up to 60 h in case of calcium alginate immobilization. This implies that the number of entrapped cells gradually decreases with increase in incubation time and so is the case with alkaline protease production (Adinarayana et al. 2005).

The results in Fig. 4, 5, 6 indicate that maximum alkaline protease production, i.e., 4200 µg/ml/min, was detectable after 48 h with polyacrylamide-entrapped cells as compared to other immobilized matrices and free cell system, Polyacrylamide was earlier successfully used for immobilization of many enzyme systems. It was used for the immobilization of cells for the production of other primary metabolites (Adinarayana et al. 2005). The results of the present investigation are in line with the findings of Abdel-Naby et al. (1998) and Kumar and Vats (2010), who has reported maximum alkaline protease production by *B. mycoides* and *B. subtilis* on polyacrylamide matrix as compared to agar, gelatin and calcium alginate. Although alkaline protease titer obtained with other matrices such as agar, calcium alginate was low, but these natural polymers were also employed for cell immobilization. They employed gel as a carrier material for the immobilization of different enzyme systems which include protease (Rao et al. 2008), β-galactosidase and penicillin acylase (Ramakrishna and Prakasham 1999). It is evident that the alkaline protease production was higher with immobilized cells (4200 µg/ml/min) than that of free cells (3450 µg/ml/min).

The semi-continuous fermentation was terminated to investigate the stability of the biocatalysts and their ability to produce alkaline protease under repeated batch cultivation conditions. Figures 4, 5 and 6 show the possibility for reuse of the polyacrylamide-immobilized cells to produce alkaline protease in semi-continuous mode. The results revealed that the amount of enzyme production with polyacrylamide-immobilized cells gradually decrease in alkaline protease titer from the first batch onward. At the same time, the gradual cell leakage from the gel was observed from the first to the ninth batch. There is no disintegration of the beads; however, a huge loss of protease activity was observed as compared to the first batch of fermentation. Thus, the repeated batch fermentation with polyacrylamide beads was successfully run for nine batches (18 days). These findings were in accordance with those obtained previously for the protease production by immobilized *Pseudomonas* sp. in polyacrylamide beads (Sankaralingam et al. 2012). The agar and calcium alginate-entrapped cells were also used for repeated batch fermentation. The behavior for these systems was similar to polyacrylamide cells. With all these systems, relatively low enzyme titer was observed when compared with polyacrylamide cells. Also, the cell leakage from the respective gels was more and gel cubes of agar disintegrated after five batches of fermentation.

Thus, polyacrlyamide was found superior to other matrices, as a maximum total protease titer (24,847 µg/ml/min) was obtained with this system which was higher than that in other reports. The present study reported that even after the immobilized cells had been in use for about 18 days, it still possessed significant alkaline protease production. It has been shown that immobilized cells were able to produce alkaline protease consistently and that they might be used for continuous alkaline protease production (Beshay 2003).

In the present investigation, the role of immobilized alkaline protease produced by *B. amyloliqefaciens* SP1 in detergent was studied. Rapid bloodstain removal was noticed with supplementation of commercially available detergents, i.e., Ariel with immobilized protease in less than 25 min. However, individual treatment of distilled water, detergent (1 % Ariel) and immobilized enzyme was not able to remove stains. There are few reports showing the use of immobilized protease as detergent additive; however, similar results were noticed with protease of *B. alveayuensis* CAS 5 (Annamalai et al. 2013).

## Conclusion

The present study reported immobilization of detergent stable, alkalophilic protease and rhizobacteria, i.e., *B. amyloliquefaciens* SP1 having multifarious plant growth-promoting traits. The highest enzyme production was recorded in cells immobilized with polyacrylamide (4200 µg/ml/min) after 48 h of incubation. Results also revealed that the amount of protease yield with immobilized cells gradually decreased from the end of the first batch due to cell leakage. However, repeated batch fermentation performed well in the case of polyacrylamide-immobilized cells that ran in nine batches. Similar results were obtained with immobilization of the enzyme. Maximum enzyme activity (5698.99 µg/ml/min) resulted when protease was immobilized in polyacrylamide matrix (10 %) as compared to calcium alginate (2829.92 µg/ml/min at 3 %), agar (2600 µg/ml/min at 2 %) and free cell system (3580 µg/ml/min). The potential application of immobilized alkaline protease of *B. amyloliquefaciens* SP1 in the detergent industry and the need of development of economic methods for improved enzyme production make whole cell and enzyme immobilization excellent alternative methods.

## References

[CR1] Abdel-Naby MA, Ismail AMS, Ahmed SA, Abdel Fattah AF (1998). Production and immobilization of alkaline protease from *Bacillus mycoides*. Bioresour Technol.

[CR2] Adinarayana K, Bezawada J, Poluri E (2005). Production of alkaline protease with immobilized cells of *Bacillus subtilis* PE-11 in various matrices by entrapment technique. AAPS Pharm Sci Tech.

[CR3] Ahmed SA, Ramadan A, Al-Domany MA, Nefisa El-Shayeb HR, Hesham Saleh SA (2010). Optimization, immobilization of extracellular alkaline protease and characterization of its enzymatic properties. Res J Agri Biol Sci.

[CR4] Annamalai N, Rajeswari MV, Balasubramanian T (2013) Extraction, purification and application of thermostable and halostable alkaline protease from *Bacillus alveayuensis* CAS 5 using marine wastes. Food Bioprod Process 434–439

[CR5] Anwar A, Qader SAU, Raiz A, Iqbal S, Azhar A (2009). Calcium alginate: a support material for immobilization of proteases from newly isolated strain of *Bacillus subtilis* KIBGE-HAS. World Appl Sci J.

[CR6] Beshay U (2003). Production of alkaline protease by *Teredinobacter turnirae* cells immobilized in Ca-alginate beads. Afr J Biotechnol.

[CR7] Demirkan E, Dincbas S, Sevinc N, Ertan F (2011). Immobilization of *B. amyloliquefaciens* α-amylase and comparison of some of its enzymatic properties with the free form. Rom Biotechnol Lett.

[CR8] Denizci AA, Kazan D, Abeln ECA, Erarslan A (2004). Newly isolated *Bacillus clausii* GMBAE 42: an alkaline protease producer capable to grow under highly alkaline conditions. J Appl Microbiol.

[CR9] Devi MK, Banu AR, Gnanaprabhal GR, Pradeep BV, Palaniswamy M (2008). Purification, characterization of alkaline protease from native isolate *Aspergillus niger* and its compatibility with commercial detergents. Indian J Sci Technol.

[CR10] Enyard CC (2008). Sigma’s non-specific protease activity assay-casein as substrate. J Vis Exp.

[CR11] Gangadharan D, Nampoothiri KM, Sivaramakrishnan S, Pandey A (2009). Immobilized bacterial α-amylase for effective hydrolysis of raw and soluble starch. Food Res Int.

[CR12] Geethanjali S, Subash A (2013). Optimization and immobilization of purified *Labeo rohita* visceral protease by entrapment method. Enzyme Res.

[CR13] Guleria S, Walia A, Chauhan A, Shirkot CK (2014) Genotypic and phenotypic diversity analysis of alkalophilic proteolytic *Bacillus* sp. associated with rhizosphere of apple trees in trans Himalayan region of Himachal Pradesh. Proc Natl Acad Sci India Sect B Biol Sci. doi:10.1007/s40011-014-0447-z

[CR14] Guleria S, Walia A, Chauhan A, Shirkot CK (2015). Purification and characterization of detergent stable alkaline protease from *Bacillus amyloliquefaciens* SP1 isolated from apple rhizosphere. J Basic Microbiol.

[CR15] Guleria S, Walia A, Chauhan A, Shirkot CK (2016). Molecular characterization of alkaline protease of *Bacillus amyloliquefaciens* SP1 involved in biocontrol of *Fusarium oxysporum*. Int J Food Microbiol.

[CR16] Jisha VN, Smitha RB, Pradeep S, Sreedevi S, Unni KN, Sajith S, Priji P, Josh MS, Benjamin S (2013). Versatility of microbial proteases. Adv Enzyme Res.

[CR17] Kotwal SM, Shankar V (2009). Immobilized invertase. Biotechnol Adv.

[CR18] Kumar R, Vats R (2010). Protease production by *Bacillus subtilis* immobilized on different matrices. New York Sci J.

[CR19] Kumar RSS, Vishwanath KS, Singh SA, Rao AGA (2006). Entrapment of α-amylase in alginate beads: single step protocol for purification and thermal stabilization. Process Biochem.

[CR20] Lamas EM, Berros RM, Balcao VM, Malcata FX (2001). Hydrolysis of whey protein by protease extracted from *Cynara cardunculus* and immobilized into highly activated supports. Enzyme Microbiol Technol.

[CR21] Lowry DH, Rosebrough NJ, Farr AL, Randall RJ (1951) Protein measurement with the Folin-phenol reagent. J Biol Chem 193:265–275 14907713

[CR22] Qureshi AS, Bhutto MA, Khushk I, Dahot MU (2011). Optimization of cultural conditions for protease production by *Bacillus subtilis* EFRL 01. Afr J Biotechnol.

[CR23] Ramakrishna SV, Prakasham RS (1999). Microbial fermentation with immobilized cells. Curr Sci.

[CR24] Rao CS, Madhavendra SS, Rao RS, Hobbs PJ, Prakasham RS (2008). Studies on improving the immobilized bead reusability and alkaline protease production by isolated immobilized *Bacillus circulans* (MTCC 6811) using overall evaluation criteria. Appl Biochem Biotechnol.

[CR25] Samia AA, Ahmed FAF (2010). Production of *Bacillus licheniformis* ATCC 21415 alkaline protease in batch, repeated batch and continuous culture. Malays J Microbiol.

[CR26] Sankaralingam S, Shankar T, Sendeshkannan K, Ramasubburayan R, Prakash S (2012). Production of Protease from *Pseudomonas* sp. by immobilization approach on different matrices. Eur. J Appl Sci.

[CR27] Tanksale A, Chandra PM, Rao M, Deshpande V (2001). Immobilization of alkaline protease from *Conidiobolus macrospores* for reuse and improved thermal stability. Biotechnol Lett.

[CR28] Wang SL, Chao CH, Liang TW, Chen CC (2009). Purification and characterization of protease and chitinase from *Bacillus cereus* TKU006 and conversion of marine wastes by these enzymes. Mar Biotechnol.

